# Effect of online Kundalini Yoga mental health of university students during Covid-19 pandemic: A randomized controlled trial

**DOI:** 10.1177/13591053231220710

**Published:** 2024-01-17

**Authors:** Tânia Brandão, Inês Martins, Ana Torres, Sónia Remondes-Costa

**Affiliations:** 1 William James Center for Research Ispa—Instituto Universitário, Portugal; 2 Departamento de Educação e Psicologia, Escola de Ciências Humanas e Socias, Universidade de Trás-os-Montes e Alto Douro, Portugal; 3 Department of Psychology and Education, Faculty of Social Sciences, University of Beira Interior, Portugal; 4 Center for Health Technology and Services Research of the Health Research Network (CINTESIS@RISE), Department of Education and Psychology, University of Aveiro, Portugal

**Keywords:** autogenic training, online, randomized controlled trial, university students, yoga

## Abstract

University students were at an increased risk for psychological distress during the COVID-19 pandemic. Using a randomized controlled trial, we examined the efficacy of an online *Kundalini* Yoga intervention on students’ psychological functioning. Healthy university students (*N* = 106) were randomly assigned to a *Kundalini* Yoga group, an active control group, or a passive control group in a 1:1:1 ratio. The experimental group attended six Yoga sessions over 6 weeks and the active control group attended to six autogenic relaxation sessions over 6 weeks. All participants completed the study protocol, which involved answering questionnaires related to psychological distress, emotion regulation, self-compassion, self-concept, spiritual well-being, and subjective happiness at three different time points: baseline, at the end of the intervention, and at 1-month follow-up. Results showed that Yoga contributed to improving self-compassion, extrinsic affect improving, and personal and communal spiritual well-being, in comparison to the control groups.

## Introduction

The mental health of university students is a growing concern in recent years (Lipson et al., 2019; Auerbach et al., 2018). This concern has been exacerbated since the spread of the coronavirus disease (COVID-19) that enforced strict social distancing measures. A recent review and meta-analysis indicated that the prevalence of depression (around 39%) and anxiety (around 36%) increased among university students during this period (Li et al., 2021). Loneliness was also a complaint with a high prevalence among college students (54.1%) (Lee et al., 2021). Thus, providing interventions to university students is crucial in reducing psychopathological symptoms and coping with daily life stress, as mental health issues can negatively impact academic outcomes (Kötter et al., 2017).

To avoid physical contact online interventions have been considered promising tools for promoting mental health (e.g. Yang et al., 2021). Also, holistic movement practices (e.g. Yoga, Tai Chi), aiming at promoting well-being and alleviating stress are considered important for university students since they provide a wide range of benefits (e.g. Winzer et al., 2018).

Yoga has its root in ancient India and is recognized as a mind-body therapy integrated into alternative medicine (Bridges and Sharma, 2017). It includes physical fitness parameters such as muscular strength, flexibility, functional mobility, balance, and aerobic capacity (e.g. Gothe and McAuley, 2016). Specifically, Kundalini Yoga is a practice that is focused on the awakening of the dormant energy that is said to be coiled at the base of the spine, known as *kundalini*. Kundalini Yoga aims to awaken and raise this energy up through the chakras, which are energy centers in the body, to achieve higher states of consciousness and spiritual enlightenment. It incorporates a range of techniques, including physical postures (asana), breathwork (pranayama), meditation, chanting (mantra), and mudras (hand gestures), to help awaken and move the kundalini energy (Pandit, 1993).

Yoga is likely to be effective in promoting mental health since it induces parasympathetic nervous system activation and suppresses sympathetic nervous system activation (Pramanik et al., 2009; Ross and Thomas, 2010). Reviews have shown that Yoga contributes to reducing depression (Bridges and Sharma, 2017), anxiety, and distress (Cramer et al., 2018; Li and Goldsmith, 2012), and can improve self-compassion, spirituality, mindfulness, self-awareness, strengthened coping mechanisms, appraisal of control, and calmness (Riley and Park, 2015). Empirical studies about the effects of Kundalini Yoga also point to improvements in perceived stress, affect, emotion regulation, resilience, fatigue (e.g. García-Sesnich et al., 2017; McMahon et al., 2021; Sarkissian et al., 2018). Previous studies that applied Yoga to university students also pointed to improvements in self-concept (Dol, 2019) and emotional intelligence (Ganpat, 2020), distress symptoms, life satisfaction, and sleep disturbances (Elstad et al., 2020). What remains to know is if Yoga delivered online is effective and in which dimensions, especially during a stressful time such as a pandemic. In a recent study (Chang et al., 2022), it was found that online Upa Yoga seemed to mitigate the pandemic’s negative impact by improving undergraduates’ mental health and well-being.

### This study

Through a randomized controlled trial (RCT), the current study aimed to examine the efficacy of a Yoga intervention delivered online compared to two control groups (one active control group—that offered six sessions of autogenic relaxation also online; and one passive control group—with no intervention) among Portuguese university students. An active control group was included to allow establishing efficacy of Yoga compared to an existing non-physical activity-based treatment (autogenic training incorporates only a specific relaxation technique emphasizing the repetition of phrases or visualizations) (Kinser and Robins, 2013). By comparing these practices, the study can shed light on their unique characteristics and elucidate the potential benefits derived from their combined physical and relaxation components. Also, a passive control group was included to allow establishing efficacy of Yoga compared to no treatment.

Based on a previous study (Chang et al., 2022) that examined the efficacy of an online Isha Upa Yoga intervention (in comparison to a passive control group only), we hypothesized that participants in the experimental group (i.e. online Yoga—*Kundalini* intervention) would experience higher improvements in both primary and secondary outcomes, in comparison to both the active control group (autogenic relaxation intervention) and the passive control group. Including an active control group will enable the comparison of the effects of Yoga with those of other approaches.

## Method

### Study design

This study follows the Holistic Movement Practices REsearch DEsign and Reporting guidelines (Vergeer et al., 2023). The study was approved by the Ethics Committee of the University of Trás-os-Montes and Alto Douro. The experimental design involved a randomized controlled trial with an experimental group (EG: Yoga) and two control groups (CG1-an active control group–Relaxation; and CG2-a passive control group—with no intervention). Participants were randomly assigned to one of the three groups (allocation ratio of 1:1:1), manually by the second author, according to the alphabetic order of the participants’ name. Allocation process was not concealed, and this was a not-blinded study due to the implicit nature of the intervention. The study included three waves of assessment: T0 (baseline), T1 (post-intervention), and T2 (1-month follow-up). The interventions occurred in October/November 2020 and data collection occurred between October 2020 and January 2021, a period in which covid-19 rate increased in Portugal and social distancing measures were employed.

### Participants

Based on the meta-analysis conducted by Harrer et al. (2019) that evaluated the effects of internet interventions for mental health in university students, a moderate treatment effect size of *g* = 0.24 was chosen (calculated using the mean effect size for depression, anxiety, and stress across studies with convenience samples included in the subgroup analyses of the meta-analysis). Using G*Power software, we calculated that a total sample size of 108 participants would be necessary to detect this effect size for psychological distress between the experimental group and the two control groups with 80% power and two-sided α = 0.05.

The inclusion criteria for this study were university students who were over 18 years of age. No specific exclusion criteria were set. A convenience sample was used—participants were recruited based on their availability and willingness to participate (not using a random selection process). Portuguese university students from School of Human and Social Sciences and School of Life and Environmental Sciences from University of Trás-os-Montes and Alto Douro were invited to participate during classes.

### Interventions

#### Experimental group: Yoga

Participants in the EG participated in six weekly, 60 minutes sessions of Yoga online via zoom, in a group-format. They were exposed to a specific type of Yoga—*Kundalini* (KY), which is a form of Yoga that involves chanting, singing, breathing exercises, and repetitive poses, to promote an elevated state of awareness (KY as taught by Yogi Bhajan). The intervention was not available during follow-up period.

The intervention was delivered by a registered Yoga teacher (certified by the International Kundalini Yoga Teacher’s Association (IKYTA) e by the Kundalini Research Institute). A standardized handout was provided to participants to enhance their understanding of the practice (available in Supplemental Appendix). Intervention sessions followed this structure: (1) chanting opening mantras; (2) warm-up breathing exercises (*Pranayama*) to promote the amount, quality, flow, and direction of vital energy in the body); (3) specific sequence of physical postures (*Kriya*) involving breath patterns, eye focus, and hand positions, to create a predictable outcome in consciousness; (4) relaxation; (5) meditation; (6) chanting closing mantras.

#### Active control group: Autogenic training

Students at the CG1 participated in six weekly, 60 minutes sessions of the autogenic training (AT) (as proposed by Johannes Schultz), also online via zoom. The intervention was delivered by the second author of the study under the supervision of the last author, that is formed and has experience in AT.

The AT is “a passive autosuggestion technique with the goal of self-produced relaxation with a minimal amount of training” (McNeil and Lawrence, 2002). It includes six mental exercises: heaviness (to reduce muscular tension, e.g. “*My legs are very heavy*”), warmth (to promote blood circulation, e.g. “*My arms are very warm*”), heart regulation (to promote awareness of heart activity, e.g. “*My heart is beating calmly and strongly*,” breathing regulation (e.g. “*My breath is calm*”), regulation of the visceral organs (to focus attention on central nervous system, e.g. “*Warmth radiates over my abdomen*”), and regulation of the temperature of the head (to promote vasoconstriction, e.g. “*My forehead is cool and relaxed*”) (McNeil and Lawrence, 2002). Additionally, guided imagery was used to promote health and personal power (protocol is available in the Supplemental Appendix).

#### Passive control group: No intervention

Participants assigned to the control group only filled out the questionnaires in the three waves of assessment.

### Measures

#### Primary outcome

Depression, anxiety, and stress were measured using the DASS-21 (Lovibond and Lovibond, 1995; Portuguese version: Pais-Ribeiro et al., 2004). DASS-21 measures the emotional states of depression (item example “*I couldn’t seem to experience any positive feeling at all*”), anxiety (item example: “*I was aware of dryness of my mouth*”), and stress (item example “*I found it hard to wind down*”) with 21 items. Each dimension has seven items scored on a 4-point Likert-type scale ranging from 0 (*did not apply to me at all*) to 3 (*applied to me very much or most of the time*). In the present study, Cronbach alfa was 0.92 for depression, 0.88 for anxiety, and 0.90 for stress.

#### Secondary outcome

Self-compassion was measured using the Self-Compassion Scale (Neff, 2003; Portuguese version: Castilho and Gouveia, 2011). It includes 26 items, divided into six dimensions: self-kindness (item example “*I try to be loving toward myself when I’m feeling emotional pain*”), self-judgment (item example “*When times are really difficult, I tend to be tough on myself*”—reversed), common humanity (item example “*When things are going badly for me, I see the difficulties as part of life that everyone goes through*”), isolation (item example “*When I think about my inadequacies, it tends to make me feel more separate and cut off from the rest of the world*”—reversed), mindfulness (item example “*When something upsets me I try to keep my emotions in balance*”), and over-identification (item example “*When I’m feeling down I tend to obsess and fixate on everything that’s wrong*” —reversed). Items are scored on a 5-point Likert-type scale ranging from 1 (*almost never*) to 5 (*almost always*). In the present study, Cronbach alfa was 0.90 for the total score of the scale.

Self-concept was measured using the Self-Concept Clinical Inventory (Vaz-Serra, 1986). It has 20 items and includes four dimensions: social acceptance (item example: “*I am usually well-accepted by others*,” self-efficacy (item example “*I usually give up on tasks when face difficulties*”), psychological maturity (item example “*I think I am tolerant with others*”), and impulsivity-activity (item example “*I am a person that likes to do what I want*”). Items are scored on a 5-point Likert-type scale ranging from 1 (*very uncharacteristic*) to 5 (*very characteristic*). In the present study, Cronbach alfa was 0.89 for the total score.

Emotion regulation was measured using the Emotion Regulation of Others and Self (Niven et al., 2011; Portuguese version; masked for review). It has 19 items, divided into four dimensions: intrinsic affect-improving (item example “*I thought of positive aspects of my situation*”), intrinsic affect-worsening (item example “*I looked for problems in my current situation*”), extrinsic affect-improving (item example “*I spent time with someone*”), and extrinsic affect-worsening (item example “*I explained to someone how they had hurt myself or others*”). Items are scored on a 5-point Likert-type scale ranging from 1 (*not at all*) to 5 (*a great deal*). In the present study, Cronbach alfa was 0.85 for intrinsic affect-improving, 0.87 for intrinsic-affect worsening, 0.92 for extrinsic affect-improving, and 0.80 for extrinsic affect-worsening.

Spiritual well-being was measured using the Spiritual Well-being Questionnaire (Gomez and Fisher, 2003; Portuguese version: Gouveia et al., 2009). It has 20 items divided into four dimensions: personal well-being (item example “*Developing a sense of identity*”), communal well-being (item example “*Developing trust between individuals*”), transcendental well-being (item example “*Developing oneness with God*”), and environmental well-being (item example “*Developing oneness with nature*”). Items are scored on a 5-point Likert-type scale ranging from 1 (*very low*) to 5 (*very high*). In the present study, Cronbach alfa was 0.90 for the total score of the scale.

Subjective happiness was measured using the Subjective Happiness Scale (Lyubomirsky and Lepper, 1999; Portuguese version: Pais-Ribeiro, 2012). It has four items and items are scored on a 7-point Likert-type scale ranging from 1 to 7. In the present study, Cronbach alfa was 0.61.

##### Sociodemographic questionnaire

Participants were asked to complete a sociodemographic questionnaire, which included questions about their sex, age, romantic relationship status (No/Yes), course of study (Psychology, Sociocultural Animation, Psychomotor Rehabilitation), academic year (first, second, third, first year master’s degree), and previous physical activity (No/Yes).

### Procedure

This study was approved by the Ethics Committee of the University of Trás-os-Montes and Alto Douro. Students were recruited from September 2020 to October 2021 through professors of three different courses: Psychology, Sociocultural Animation, and Psychomotor Rehabilitation (in person and via zoom classes). The students were briefed about the study’s purpose and the Yoga and Relaxation practices involved. Confidentiality was ensured. Participation in the study was voluntary. After that, the Yoga and Relaxation sessions were scheduled according to the students’ classes to facilitate their participation. Students were randomly assigned to one of the three groups. After that, students were contacted via email to fill out the questionnaire of the baseline wave of assessment, to inform them about the schedule of their sessions, and to provide the zoom password to have access to the sessions. At the end of the intervention and at the follow-up they were contacted again by email to fill out again the questionnaires (including the passive control group). The researcher kept personal notes about session attendance (i.e. present vs absent). Compliance with the session ingredients was not measured. The program proceeded as planned, thus no differences existed between the proposed program and the delivered program.

### Data analysis

Data were analyzed using SPSS software (version 28). The homogeneity of the groups (to assure the success of the randomization) in terms of sociodemographic variables and outcomes at baseline was examined using analysis of variance (ANOVA) for global scores and multivariate analysis of variance (MANOVA) for subscales.

To evaluate the effect of Yoga on primary and secondary outcomes the General Linear Model—ANOVA with repeated measures—was used. Effects of time (intervention effect), effects of group, and time × group interactions (delivery format effect) across the three waves of assessments were examined. Assumption of sphericity was examined using the Mauchly’s Test of Sphericity, with a non-significant result allowing to assume sphericity. When sphericity was not assumed (*p* < 0.05), the Greenhouse-Geisser correction was used. All tests were two-tailed and *p* < 0.05 was considered statistically significant.

## Results

### Participants’ baseline characteristics

A total of 133 students agreed to participate and were randomly assigned to one of three groups: 45 students were assigned to the experimental group (EG), while 44 were assigned to each of the two control groups (CG1 and CG2). During the study, 17 students from the EG and ten students from the CG1 withdrew from the study (they did not complete the baseline questionnaires after randomization and did not attend any sessions—main reasons due to scheduling incompatibility). The final number of participants included in the analysis was 106—all these participants responded to the baseline questionnaires and successfully completed the intervention; 28 in the EG, 34 in the CG1, and 44 in the CG2 (The CONSORT diagram for the study is shown in Figure S1 in Supplemental File). The dropout rate was 20%. Baseline characteristics of the sample (*n* = 106) are presented on Table 1. Table 2 presents baseline mean comparisons between groups.

**Table 1.** Sociodemographic characteristics of the three groups.

Variables	EG (*n* = 28)	CG1 (*n* = 34)	CG2 (*n* = 44)	Total sample (*N* = 106)	Differential analysis
	*M* (SD) or *n* (%)	*M* (SD) or *n* (%)	*M* (SD) or *n* (%)	*M* (SD) or *n* (%)	
Sex					
Female	24 (85.7)	32 (94.1)	40 (90.9)	96 (90.6)	χ² = 1.28, *p* = 0.527
Male	4 (14.3)	2 (5.9)	4 (9.1)	10 (9.4)	
Age	21.46 (5.70)	21.35 (4.64)	20.52 (3.75)	21.04 (4.59)	*F* (2,103) = 0.47, *p* = 0.624
Romantic relationship					
No	12 (42.9)	23 (67.6)	22 (50)	67 (53.8)	χ² = 4.23, *p* = 0.121
Yes	16 (57.1)	11 (32.4)	22 (50)	48 (46.2)	
Course					
Psychology	17 (60.7)	22 (64.7)	25 (56.9)	64 (60.4)	χ^2^ = 4.38, *p* = 0.625
Sociocultural animation	2 (7.1)	1 (2.9)	7 (15.8)	10 (9.4)	
Psychomotor rehabilitation	9 (32.2)	11 (32.4)	12 (27.3)	32 (30.2)	
Year					
First	4 (14.3)	4 (11.8)	10 (22.7)	18 (17.0)	χ^2^ = 2.32, *p* = 0.888
Second	3 (10.7)	5 (14.7)	5 (11.4)	13 (12.3)	
Third	18 (64.3)	22 (64.7)	24 (54.5)	64 (60.4)	
First year master’s degree	3 (10.7)	3 (8.8)	5 (11.4)	11 (10.4)	
Previous physical activity					
No	23 (82.1)	15 (44.1)	18 (40.9)	56 (52.8)	χ^2^ = 13.20, *p* < 0.001
Yes	5 (17.9)	19 (55.9)	26 (59.1)	50 (47.2)	

**Table 2.** Baseline mean comparisons between the three groups.

Variables	EG (*n* = 28)	CG1 (*n* = 34)	CG2 (*n* = 44)	Mean differences
	*M* (SD)	*M* (SD)	*M* (SD)	
Primary outcome				
Depression	5.43 (4.39)	6.41 (5.67)	5.82 (5.15)	*Z*(2) = 0.29, *p* = 0.748
Anxiety	5.32 (4.60)	5.38 (5.11)	4.98 (5.01)	*Z*(2) = 0.08, *p* = 0.927
Stress	8.68 (5.39)	9.09 (5.39)	8.18 (4.93)	*Z*(2) = 0.30, *p* = 0.745
Secondary outcomes				
*Self-compassion*				
Global score	3.18 (0.70)	3.14 (0.47)	3.11 (0.58)	*Z*(2) = 0.12, *p* = 0.890
*Self-concept*				
Global score	75.46 (11.57)	72.85 (9.62)	75.91 (10.23)	*Z*(2) = 90, *p* = 0.409
*Emotion regulation*				
Extrinsic affect-improving	3.98 (0.99)	3.78 (0.85)	3.92 (0.81)	*Z*(2) = 46, *p* = 0.631
Extrinsic affect-worsening	1.30 (0.63)	1.40 (0.61)	1.31 (0.74)	*Z*(2) = 0.27, *p* = 0.761
Intrinsic affect-improving	3.35 (0.92)	3.17 (0.79)	3.28 (0.80)	*Z*(2) = 0.36, *p* = 0.696
Intrinsic affect-worsening	1.56 (0.54)	1.86 (1.07)	1.59 (0.80)	*Z*(2) = 0.94, *p* = 0.267
*Spiritual well-being*				
Global score	13.50 (3.48)	14.35 (2.60)	14.05 (2.79)	*Z*(2) = 0.66, *p* = 0.520
Personal	3.51 (0.93)	3.75 (0.68)	3.71 (0.71)	*Z*(2) = 0.86, *p* = 0.426
Communal	3.82 (0.96)	4.06 (0.60)	4.04 (0.65)	*Z*(2) = 1.00, *p* = 0.371
Environmental	3.44 (1.03)	3.64 (0.89)	3.67 (0.95)	*Z*(2) = 0.53, *p* = 0.588
Transcendental	2.73 (1.25)	2.91 (1.14)	2.62 (1.13)	*Z*(2) = 0.59, *p* = 0.556
*Subjective happiness*				
Global score	4.82 (0.94)	**4.51 (0.92)**	**5.05 (0.85)**	***Z*(2) = 0.345, *p* = 0.035**

For global scores we ran ANOVAS; for subscales of the same scale, we ran MANOVAS. Bold indicates post hoc Bonferroni comparisons.

No differences were found between groups for all outcome variables, exception being for subjective well-being. For subjective well-being, participants in the passive control group presented significantly higher levels (*M* = 5.05; *SD* = 0.85) than the active control group (*M* = 4.51; *SD* = 0.82)—no differences were found between the experimental group and the control groups. Also, a significant association was found between type of group and prior physical activity (χ^2^ = 13.20, *p* < 0.001), suggestion that being in the control group is associated with a higher likelihood of practicing physical exercise (52%), while being in the experimental is associated with a higher likelihood of not practicing physical exercise (10%) (moderation analyses according to previous physical activity are reported on Supplemental File Table S3—no significant interaction were found).

### Effects of intervention

#### Primary outcomes

Mauchly’s tests were non-significant for all primary outcomes. Results are presented in Table 3. For depression, the results showed a significant main effect of measurement time (*F*(2) = 11.65, *p* < 0.001, *η_p_*^2^ = 0.102), reflecting a decreased in depressive symptoms over time. Pairwise comparisons showed that the decline in depression was significant from baseline to post-intervention (IJ mean difference = 1.99, *p* < 0.001, 95% CI [0.921, 3.05]) and from baseline to follow-up (IJ = 1.22, *p* < 0.05, 95% CI [0.163, 2.27]). Neither the group effect (*F*(2) = 0.34, *p* = 0.714, *η_p_*^2^ = 0.007) nor the interaction effect between measurement time and group were significant (*F*(4) = 1.94, *p* = 0.109, *η_p_*^2^ = 0.036).

**Table 3.** Primary and secondary outcomes results.

Variables	Experimental group			Active control group			Passive control group			Time effect	Group effect	Time × Group effect
	T1	T2	T3	T1	T2	T3	T1	T2	T3			
Depression	5.43 (4.39)	4.18 (4.36)	3.75 (4.19)	6.41 (5.67)	3.09 (3.25)	4.82 (4.15)	5.82 (5.15)	4.43 (4.82)	5.43 (5.11)	***F* = 11.65, *p* < 0.001**	*F* = 0.34, *p* = 0.714	*F* = 1.94, *p* = 0.109
Anxiety	5.32 (4.60)	4.79 (4.41)	3.64 (4.32)	5.38 (5.11)	3.24 (2.72)	4.44 (3.52)	4.98 (5.02)	4.66 (5.34)	4.75 (5.08)	***F* = 4.75, *p* = 0.01**	*F* = 0.11, *p* = 0.893	*F* = 2.34, *p* = 0.056
Stress	8.68 (5.39)	6.71 (4.75)	6.61 (4.26)	9.09 (5.39)	6.85 (3.95)	7.79 (4.75)	8.18 (4.93)	7.23 (4.84)	8.30 (5.24)	***F* = 6.95, *p* < 0.01**	*F* = 0.28, *p* = 0.760	*F* = 1.59, *p* = 0.179
*Self-compassion*												
Global score	3.18 (0.70)	3.49 (0.71)	3.44 (0.56)	3.14 (0.47)	3.29 (0.67)	3.16 (0.71)	3.11 (0.58)	3.14 (0.61)	3.24 (0.71)	***F* = 8.22, *p* < 0.001**	*F* = 1.12, *p* = 0.330	***F* = 2.94, *p* < 0.05**
*Self-concept*												
Global score	75.46 (11.57)	78.89 (8.57)	80.25 (8.28)	72.85 (9.62)	76.91 (8.07)	75.71 (8.86)	75.91 (10.23)	77.05 (8.48)	77.30 (8.35)	***F* = 9.56, *p* < 0.001**	*F* = 1.14, *p* = 0.324	*F* = 1.31, *p* = 0.271
*Emotion regulation*												
Extrinsic-affect improving	3.98 (0.87)	4.44 (0.52)	3.84 (0.81)	3.78 (0.85)	3.85 (0.73)	3.45 (1.10)	3.92 (0.81)	3.72 (0.82)	3.67 (0.87)	***F* = 10.44, *p* < 0.001**	*F* = 2.57, *p* = 0.082	***F* = 3.71, *p* < 0.01**
Extrinsic-affect worsening	1.30 (0.47)	1.21 (0.30)	1.21 (0.34)	1.39 (0.60)	1.28 (0.35)	1.32 (0.62)	1.31 (0.74)	1.19 (0.33)	1.34 (0.53)	*F* = 1.85, *p* = 0.168	*F* = 0.413, *p* = 0.663	*F* = 0.422, *p* = 0.757
Intrinsic-affect improving	3.35 (0.92)	3.67 (0.79)	3.27 (0.88)	3.14 (0.79)	3.25 (0.78)	3.14 (0.87)	3.28 (0.80)	3.14 (0.83)	3.17 (0.92)	*F* = 2.50, *p* = 0.085	*F* = 1.23, *p* = 0.328	*F* = 2.18, *p* = 0.072
Intrinsic-affect worsening	1.56 (0.54)	1.47 (0.67)	1.39 (0.65)	1.84 (1.06)	1.57 (0.79)	1.74 (1.07)	1.59 (0.80)	1.48 (0.81)	1.58 (0.84)	*F* = 2.18, *p* = 0.120	*F* = 0.97, *p* = 0.384	*F* = 0.656, *p* = 0.611
*Spiritual well-being*												
Personal	3.51 (0.93)	3.92 (0.78)	4.06 (0.69)	3.75 (0.68)	3.81 (0.62)	3.71 (0.79)	3.71 (0.71)	3.81 (0.75)	3.80 (0.80)	***F* = 4.86, *p* < 0.01**	*F* = 0.20, *p* = 0.817	***F* = 2.78, *p* < 0.05**
Communal	3.82 (0.96)	4.28 (0.63)	4.21 (0.55)	4.06 (0.60)	4.13 (0.61)	4.15 (0.58)	4.04 (0.65)	3.97 (0.73)	3.96 (0.77)	*F* = 2.57, *p* = 0.084	*F* = 0.12, *p* = 0.887	***F* = 2.67, *p* < 0.05**
Environmental	3.44 (1.03)	3.58 (1.02)	3.59 (1.07)	3.64 (0.89)	3.77 (0.84)	3.61 (0.94)	3.67 (0.95)	3.72 (1.01)	3.65 (1.12)	*F* = 0.591, *p* = 0.541	*F* = 0.606, *p* = 0.547	*F* = 0.225, *p* = 0.912
Transcendental	2.73 (1.25)	3.10 (1.33)	3.16 (1.44)	2.91 (1.14)	2.77 (1.11)	2.71 (1.06)	2.62 (1.13)	2.67 (1.24)	2.66 (1.30)	*F* = 0.599, *p* = 0.521	*F* = 0.27, *p* = 0.768	
*Subjective happiness*												
Global score	4.82 (0.94)	4.79 (0.93)	4.88 (0.83)	4.51 (0.92)	4.75 (0.85)	4.65 (1.05)	5.05 (0.85)	4.91 (0.96)	4.92 (0.89)	*F* = 0.107, *p* = 0.875	*F* = 0.88, *p* = 0.420	*F* = 1.93, *p* = 0.117

Bold represent significant effects.

For anxiety, the results showed a significant main effect of measurement time (*F*(2) = 4.75, *p* = 0.01, *η_p_*^2^ = 0.044). Pairwise comparisons showed that the decline in anxiety was significant from baseline to post-intervention (IJ = 1.00, *p* < 0.05, 95% CI [0.026, 1.98]) and from baseline to follow-up (IJ = 0.95, *p* < 0.05, 95% CI [0.084, 1.81]). Neither the group effect (*F*(2) = 0.11, *p* = 0.893, *η_p_*^2^ = 0.002) nor the interaction effect between measurement time and group were significant (*F*(4) = 2.34, *p* = 0.056, *η_p_*^2^ = 0.044).

For stress, the results showed a significant main effect of measurement time, *F*(2) = 6.95, *p* < 0.01, *η_p_*^2^ = 0.063, reflecting a decreased in stress symptoms over time. Pairwise comparisons showed that the decline in stress was significant only from baseline to post-intervention (IJ = 1.55, *p* < 0.01, 95% CI [0.494, 2.61]) and from baseline to follow-up (IJ = 1.08, *p* < 0.05, 95% CI [0.026, 2.14]). Again, neither the group effect (*F*(2) = 0.28, *p* = 0.760, *η_p_*^2^ = 0.005) nor the interaction effect between measurement time and group were significant (*F*(4) = 1.59, *p* = 0.179, *η_p_*^2^ = 0.030) (see Figure S1—Supplemental Appendix).

#### Secondary outcomes

For secondary outcomes, Mauchly’s tests were non-significant for some variables namely: self-concept, intrinsic affect improving, intrinsic affect worsening, communal well-being, environmental well-being, transcendental well-being, and subjective happiness—in these cases Greenhouse-Geisser correction was used.

For self-compassion, the results showed a significant main effect of measurement time (*F*(2) = 8.22, *p* < 0.001, *η_p_*^2^ = 0.074) reflecting an increase in self-compassion only from baseline to post-intervention (IJ = 0.165, *p* < 0.01, 95% CI [0.056, 2.74]) and from baseline to follow-up (IJ = 0.133, *p* < 0.01, 95% CI [0.027, 239]). The group effect was not significant (*F*(2) = 1.12, *p* = 0.330, *η_p_*^2^ = 0.021) but interaction effect between measurement time and group was significant (*F*(4) = 2.94, *p* < 0.05, *η_p_*^2^ = 0.054). Pairwise comparisons showed that the increase in self-compassion was significant only for the participants in the experimental group (IJ = 0.312, *p* < 0.01, 95% CI [0.104, 0.520] from baseline to post-intervention; and IJ = 0.258, *p* < 0.01, 95% CI [0.055, 0.451] from baseline to follow-up).

For self-concept, the results showed a significant main effect of measurement time (*F*(1.70) = 9.56, *p* < 0.001, *η_p_*^2^ = 0.085), reflecting an increase in self-concept only from baseline to post-intervention (IJ = 2.88, *p* < 0.01, 95% CI [0.756, 4.99]) and from baseline to follow-up (IJ = 3.01, *p* < 0.01, 95% CI [0.965, 5.05]). Neither the group effect (*F*(2) = 1.14, *p* = 0.324, *η_p_*^2^ = 0.022) nor the interaction effect between measurement time and group were significant, *F*(3.39) = 1.31, *p* = 0.271, *η_p_*^2^ = 0.025.

In terms of emotion regulation: for extrinsic affect improving the results showed a significant main effect of measurement time (*F*(2) = 10.44, *p* < 0.001, *η_p_*^2^ = 0.093), reflecting an increase from baseline to follow-up (IJ = 0.242, *p* < 0.01, 95% CI [0.048, 0.436]) but a decrease from post-intervention to follow-up (IJ = −0.350, *p* < 0.01, 95% CI [−0.547, −0.153]). The group effect was not significant (*F*(2) = 2.57, *p* = 0.082, *η_p_*^2^ = 0.048) but the interaction effect between measurement time and group was (*F*(4) = 3.71, *p* < 0.01, *η_p_*^2^ = 0.068). Pairwise comparisons showed that extrinsic affect improving improved significantly only for the participants in the experimental group (IJ = 0.458, *p* < 0.01, 95% CI [0.112, 0.804] from baseline to post-intervention); but it decreased significantly for these participants from post-intervention to follow-up (IJ = −0.601, *p* < 0.001, 95% CI [−0.976, −0.226]). For participants in the active control group extrinsic affect improving decreased significantly from post-intervention to follow-up (IJ = −0.404, *p* < 0.05, 95% CI [−0.749, −0.059]).

For extrinsic affect worsening time effects were not significant (*F*(1.66) = 1.85, *p* = 0.168, *η_p_*^2^ = 0.018) as well as group effects (*F*(2) = 0.41, *p* = 0.663, *η_p_*^2^ = 0.008) and time × group effects (*F*(3.31) = 0.422, *p* = 0.757, *η_p_*^2^ = 0.008 for time × group effects).

For Intrinsic affect improving, time effects (*F*(2) = 2.50, *p* = 0.085, *η_p_*^2^ = 0.024), group effects (*F*(2) = 1.13, *p* = 0.328, *η_p_*^2^ = 0.021) and group*time effects were non-significant (*F*(4) = 2.18, *p* = 0.072, *η_p_*^2^ = 0.041). The same happened for Intrinsic affect worsening (*F*(1.85) = 2.18, *p* = 0.120, *η_p_*^2^ = 0.021 for time effects, *F*(2) = 0.97, *p* = 0.384, *η_p_*^2^ = 0.018 for group effects, and *F*(3.70) = 0.656, *p* = 0.611, *η_p_*^2^ = 0.013 for time × group effects) (see Figure S2 Supplemental Appendix).

In terms of spiritual well-being: for personal well-being the results showed a significant main effect of measurement time. (*F*(2) = 4.86, *p* < 0.01, *η_p_*^2^ = 0.045), reflecting an increase from baseline to post-intervention (IJ = 0.191, *p* < 0.05, 95% CI [0.013, 0.369]) and from baseline to follow-up (IJ = 0.200, *p* < 0.05, 95% CI [0.012, 0.388]). The group effect was not significant (*F*(2) = 0.12, *p* = 0.887, *η_p_*^2^ = 0.002) but the interaction effect between measurement time and group was significant (*F*(4) = 2.78, *p* < 0.05, *η_p_*^2^ = 0.051). Pairwise comparisons showed that personal well-being improved significantly only for the participants in the experimental group (IJ = 0.414, *p* < 0.05, 95% CI [0.073, 0.755] from baseline to post-intervention). For communal well-being, time effects (*F*(1.83) = 2.57, *p* = 0.084, *η_p_*^2^ = 0.024) and group effects (*F*(2) = 0.67, *p* = 0.547, *η_p_*^2^ = 0.012) were non-significant but group × time effects were (*F*(3.67) = 2.67, *p* < 0.05, *η_p_*^2^ = 0.049). Pairwise comparisons showed that only for participants from the experimental group communal well-being increased from baseline to post-intervention (IJ = 0.46, *p* < 0.01, 95% CI [0.109, 0.806]) and from baseline to follow-up (IJ = 0.39, *p* < 0.05, 95% CI [0.010, 0.762]).

For environmental well-being, results showed that time effects (*F*(1.84) = 0.591, *p* = 0.541, *η_p_*^2^ = 0.006), group effects (*F*(2) = 0.27, *p* = 0.768, *η_p_*^2^ = 0.005) and time*group effects *F*(3.67) = 0.225, *p* = 0.912, *η_p_*^2^ = 0.004 for time × group effects).The same happened for transcendental well-being (*F*(1.66) = 0.599, *p* = 0.521, *η_p_*^2^ = 0.066 for time effects, *F*(2) = 0.88, *p* = 0.420, *η_p_*^2^ = 0.017 for group effects, and *F*(3.33) = 1.92, *p* = 0.121, *η_p_*^2^ = 0.036 for time × group effects).

Also, for subjective happiness, time effects (*F*(1.76) = 0.107, *p* = 0.875, *η_p_*^2^ = 0.001), group effects (*F*(2) = 1.46, *p* = 0.236, *η_p_*^2^ = 0.028) and group*time effects were non-significant (*F*(3.51) = 1.93, *p* = 0.117, *η_p_*^2^ = 0.034) (see Figure S3 Supplemental Appendix).

## Discussion

Previous studies have suggested that Yoga is effective in reducing depression, anxiety, distress, and sleep disturbances, and in improving self-concept, life satisfaction, and emotional intelligence (e.g. Bridges and Sharma, 2017; Cramer et al., 2018; Elstad et al., 2020; Ganpat, 2020; Li and Goldsmith, 2012). However, there has been a limited assessment of the effects of online Yoga on university students, particularly during periods of heightened stress such as a pandemic. It is worth noting that a recent study conducted by Chang et al. (2022) found that online Isha Upa Yoga was effective in enhancing the mental health and overall well-being of university students amidst the challenges brought about by the COVID-19 pandemic.

In this RCT, a structured online Yoga—*Kundalini* intervention with six sessions offered to university students during the pandemic COVID-19 was implemented and tested, in comparison to an active control group (a structured online autogenic training with six sessions) and a passive group control. While all groups reported favorable improvements in most dimensions at the end of the intervention, findings showed that participation in the Yoga group contributed to producing significant effects on participants’ psychological functioning. Specifically, participants reported statistically significant improvement in self-compassion, emotion regulation (in terms of extrinsic affect improving), and spiritual well-being (personal and communal well-being), in comparison to both control groups. Additionally, these effects remained significant 1 month later, suggesting that they can produce long-term effects. These results are in accordance with previous studies that showed improvements in the psychological functioning of university students after participating in Yoga interventions (e.g. Chang et al., 2022; Elstad et al., 2020; Ganpat, 2020). Also, there results confirmed the benefits of Yoga– Kundalini found in previous studies in terms of stress, affect, and emotion regulation (e.g. McMahon et al., 2021; Sarkissian et al., 2018).

Our results suggested that the Yoga intervention (and in some cases, the online autogenic training) implemented can be effective in improving students’ self-compassion, emotion regulation, and spiritual well-being during stressful times. As pointed out by Chang et al. (2022), these types of interventions can be effective due to their brevity, affordability (free), and virtuality. Indeed, a six-session intervention allowed to improve different dimensions of university students’ psychological functioning as discussed below.

Improvements in self-compassion are in line with previous studies (Gorvine et al., 2019; Mathad et al., 2017). Indeed, self-compassion has been found to be a mechanism of change in Yoga interventions since it is a quality that results from contemplative practices (Riley and Park, 2015). Spiritual well-being (personal and communal) also improved in the experimental group. Indeed, a recent systematic review has suggested that Yoga practice seems to be positively linked to various aspects of spirituality (Csala et al., 2021).

It is important to note two aspects of our findings. First, participants in the active control group (i.e. online autogenic training) also experienced a marginally significant reduction in anxiety levels from baseline to post-intervention. Previous studies have shown that autogenic training can be effective in reducing psychological morbidity (e.g. Atkins and Hayes, 2019; Kulmatycki and Boroń-Krupińska, 2019) since it allows one to deal with stressful events and negative emotions (Kulmatycki and Boroń-Krupińska, 2019) by switching off the stressful fight-flight mechanism and restoring psychophysical relaxation (Klott, 2013). However, our results seem to suggest that Yoga may be more effective in terms of long-term effects.

Also, in terms of emotion regulation (namely, in terms of extrinsic affect improving), participants in the experimental group experienced a significant increase in this dimension from baseline to post-intervention but then they experienced a significant decrease from post-intervention to 1-month follow-up (this later decrease was also experienced by the participants in the active control group). While previous studies have shown that Yoga is effective in reducing negative emotions, improving positive emotions, and improving emotion regulation (Daly et al., 2015; Kahya and Raspin, 2017; Park et al., 2020), our results seem to suggest that these effects occur during the practice of Yoga, reducing when this practice ends. Other possible explanation may be related to the fact that this dimension of emotion regulation focuses on improving others’ affect and with the practice of Yoga participants learn to focus more on their own emotional states.

Our findings suggest that online Yoga can be considered a useful tool to promote the self-compassion, emotion regulation and spiritual well-being in university students not only during stressful times (such as the COVID-19 pandemic) but also as a wider strategy during normative times (e.g. final exams and dissertation defenses) through formative years.

### Limitations and future research

This study has some limitations. First, it includes a small sample size. Thus, a larger RCT should be conducted to verify the results obtained. Second, most of the participants were women; thus, future studies should include a more heterogenous sample, including more men. Third, the randomization process was not performed by an independent third which can lead to some randomization biases. Also, participants’ baseline assessment was performed after randomization (but before any intervention) which can lead to some biases since they were informed about the assigned group. Additionally, it should be noted that the use of per-protocol analysis can also introduce biases and limit the generalizability of the results to the wider population, as it excludes participants who did not strictly adhere to the protocol, which may not reflect the real-world setting where adherence to treatment is often suboptimal. Thus, future studies should include intent-to-treat analyses.

Fourth, we only collected quantitative data with self-report questionnaires. Also, depression, anxiety, and stress were measured using the DASS-21 - it is possible that using separate measures for each construct could provide more detailed information about the specific areas of emotional distress that are being impacted by the intervention. To gain a more comprehensive understanding of the experiences of participants and to identify helpful and unhelpful factors related to this type of intervention and mode of delivery, qualitative studies are necessary. Furthermore, due to the small sample size, conducting mediators’ analyses to better comprehend the mechanisms of change underlying this specific intervention was not feasible. Also, we only included one follow-up (1 month). A longer follow-up would be essential to examine if the effects of online Yoga sustain over time (for those who maintain their practice and for those who did not). Future studies should also assess the efficacy of online Yoga (including different types of Yoga) within other contexts/samples - the present study has investigated the effects exclusively among university students thus it is important to acknowledge that our findings and conclusions are limited to this specific age group and demographic. The university student population possesses distinct characteristics, including academic stress, lifestyle, and daily routines, which may impact the outcomes of Kundali yoga practice differently compared to other age groups within society. Therefore, we emphasize that the conclusions drawn from this study are specific to university students and may not be generalized to other age groups. Finally, another limitation of the study is that the frequency of the Yoga sessions, which occurred once a week, may not have been sufficiently frequent to observe significant changes in university students’ mental health.

## Supplemental Material

sj-docx-1-hpq-10.1177_13591053231220710 – Supplemental material for Effect of online Kundalini Yoga mental health of university students during Covid-19 pandemic: A randomized controlled trial Supplemental material, sj-docx-1-hpq-10.1177_13591053231220710 for Effect of online Kundalini Yoga mental health of university students during Covid-19 pandemic: A randomized controlled trial by Tânia Brandão, Inês Martins, Ana Torres and Sónia Remondes-Costa in Journal of Health Psychology
