# Supplementary material for: Effect of online Kundalini Yoga mental health of university students during Covid-19 pandemic: A randomized controlled trial
Source: J Health Psychol. 2024 Jan 17;29(6):567–80. doi: 10.1177/13591053231220710 (PMC11075415; doi:10.1177/13591053231220710)

**Supplementary Appendix**

**Table S1**

*Analysis of DASS according to cut-off scores.*

|  | Normal | Mild | Moderate/Severe |
| --- | --- | --- | --- |
| **Experimental Group - Yoga** |  |  |  |
| Depression baseline | 85.7% | 10.7% | 3.6% |
| Depression Post-intervention | 96.4% | 3.6% | - |
| Depression FU | 89.3% | 3.6% | 7.1% |
| Anxiety baseline | 75% | 7.1% | 17.9% |
| Anxiety Post-intervention | 75% | 7.1% | 17.9% |
| Anxiety FU | 82.1% | 7.1% | 10.7% |
| Stress baseline | 78.6% | 14.3% | 7.1% |
| Stress Post-intervention | 92.9% | 3.6% | 3.6% |
| Stress FU | 96.4% | 3.6% | - |
| **Active Control Group** |  |  |  |
| Depression baseline | 70.6% | 8.8% | 20.6% |
| Depression Post-intervention | 94.1% | 5.9% | - |
| Depression FU | 82.4% | 14.7% | 2.9% |
| Anxiety baseline | 76.5% | 2.9% | 20.6% |
| Anxiety Post-intervention | 88.2% | 11.8% | - |
| Anxiety FU | 79.4% | 14.7% | 5.9% |
| Stress baseline | 85.3% | 8.8% | 5.9% |
| Stress Post-intervention | 97.1% | 2.9% | - |
| Stress FU | 88.2% | 8.8% | 2.9% |
| **Passive Control Group** |  |  |  |
| Depression baseline | 81.8% | 9.1% | 9.1% |
| Depression Post-intervention | 86.4% | 6.8% | 6.8% |
| Depression FU | 81.8% | 6.8% | 11.4% |
| Anxiety baseline | 75.0% | 6.8% | 18.2% |
| Anxiety Post-intervention | 79.5% | 4.5% | 15.9% |
| Anxiety FU | 75.0% | 9.1% | 15.9% |
| Stress baseline | 84.1% | 13.6% | 2.3% |
| Stress Post-intervention | 88.6% | 9.1% | 2.3% |
| Stress FU | 86.4% | 11.4% | 2.3% |

*Note*. Depression cut-off scores: normal 0-9; mild 10-13; moderate/severe +14. Anxiety cut-off scores: normal 0-7; mild 8-9; moderate/severe +10. Stress cut-off scores: normal 0-14; mild 15-18; moderate/severe +19.

**Table S2**

*Cronbach’s Alpha Coefficients for Subscales of Study Variables.*

| **Scale** | **Subscale** | **Alpha** |
| --- | --- | --- |
| Self-Compassion Scale | Self-kindness | .85 |
|  | Self-judgement | .88 |
|  | Common humanity | .78 |
|  | Isolation | .80 |
|  | Mindfulness | .81 |
|  | Over-identification | .82 |
| Self-Concept Clinical Inventory | Social acceptance | .78 |
|  | Self-efficacy | .74 |
|  | Psychological maturity | .62 |
|  | Impulsivity-activity | .63 |
| Spiritual Well-being Questionnaire | Personal well-being | .87 |
|  | Communal well-being | .88 |
|  | Transcendental well-being | .94 |
|  | Environmental well-being | .94 |

**Table S3**

*Interaction effects with previous physical activity.*

| **Variables** | Time*Group*Physical activity |
| --- | --- |
|  |  |
| Depression | F = .99, p = .403 |
| Anxiety | F = 1.46, p = .231 |
| Stress | F = 2.17, p = .096 |
| ***Self-compassion*** |  |
| Global score | F = 1.38, p = .254 |
| ***Self-concept*** |  |
| Global score | F = .99, p = .403 |
| ***Emotion Regulation*** |  |
| Extrinsic-affect improving | F= .32, p = .314 |
| Extrinsic-affect worsening | F = .44, p = .726 |
| Intrinsic-affect improving | F= .93, p = .437 |
| Intrinsic-affect worsening | F = .38, p = .768 |
| ***Spiritual Well-being*** |  |
| Personal | F = .33, p = .807 |
| Communal | F = .98, p = .407 |
| Environmental | F= .64, p = .593 |
| Transcendental | F= 1.17, p = .325 |
| ***Subjective Happiness*** |  |
| Global score | F = 1.19, p = .319 |

1. **STRUCTURE OF KUNDALINI YOGA CLASSES**

The yoga class is divided into six parts:

**1) Initial tuning mantras**

ONG NAMO GURUDEV NAMO (Repeat three times)

AAD GUREH NAMEH, JUGAD GUREH NAMEH,

SAT GUREH NAMEH, SIRI GURU DEV NAMEH (Repeat three times)

**2) Warm-up exercises and/or Pranayama (breathing exercises to stimulate energy circulation in the body)**

**3) Kriya (set of physical postures or asanas)**

**4) Relaxation**

**5) Kundalini Yoga Medition**

**6) Closing Mantras of the Class**

Eternal Sun (Repeat twice)

SAT NAM (Repeat three times)

1. **PROTOCOL FOR SCHULTZ'S AUTOGENIC TRAINING (AT) RELAXATION SESSIONS:**

**Location** for conducting relaxation sessions should be quiet and free of noise to avoid external distractions. To facilitate relaxation, the temperature should be comfortable, lighting should be soft, and clothing should be comfortable.

**Relaxation position** - Sitting in a chair in a relaxed manner, with arms and legs uncrossed (coachman's position), hands resting on the legs facing downwards. The most important thing is that the position should be comfortable.

**Relaxation Session Planning**

**1st Stage – Beginning of Relaxation**

"To start the relaxation, I ask you to position yourselves in a relaxed and comfortable position (sitting) with your palms facing down, to concentrate on the instructions that will be given, and when you feel ready, gently close your eyes, closing the "windows" to the outside and opening the "windows" of the interior. At this moment, breathing will be the vehicle for the process of relaxation, relaxation, and energizing of your system (body-mind). Try to make a nasal breath. Take a deep breath through your nose, filling your abdomen and feeling the weight of the air. Visualize the air entering through the nostrils and being transported to the lungs, bringing relaxation, well-being, health, energy, and vitality to every cell in your system. Hold the air briefly within your body to expand the energy within you, and exhale deeply again through the nose, letting go of all worries, fears, and anxieties, feeling yourself become lighter and looser with each exhale, releasing everything that weighs you down. While mentally internalizing the phrase "I feel completely calm and relaxed," maintain a deep, nasal, and harmonious breath. " (Repeat this instruction 3 times). Breathing should be deep, nasal, and silent.

**2nd Stage - Body Awareness**

Exercise of Weight (muscle relaxation is given)

Repeat 5 times internally to yourself: "My arms are pleasantly heavy and relaxed"

(Pause - a moment of silence is allowed)

Repeat 1 time internally to yourself: "I am completely calm and relaxed"

(Pause - a moment of silence is allowed)

Repeat 5 times internally to yourself: "My legs are pleasantly heavy"

(Pause - a moment of silence is allowed)

Repeat 1 time internally to yourself: "I am completely calm and relaxed"

(Pause - a moment of silence is allowed)

Maintain awareness of calm, nasal, and silent breathing.

→ Here the participant focuses on the sensation of weight on a specific part of the body until they can generalize it to the rest of the body.

Exercise of warmth (inducing vasodilation)

Repeat to yourself 5 times: "My arms are pleasantly warm"

(Pause - allow a moment of silence)

Repeat to yourself 1 time: "I am completely calm and relaxed"

(Pause - allow a moment of silence)

Repeat to yourself 5 times: "My legs are pleasantly warm"

(Pause - allow a moment of silence)

Repeat to yourself 1 time: "I am completely calm and relaxed"

(Pause - allow a moment of silence)

Maintain awareness of calm, nasal and silent breathing.

→ Here the participant focuses on the sensation of warmth on a specific part of the body until they can generalize it to the rest of the body.

Heart Exercise (cardiac regulation is given)

Repeat to yourself 5 times: "My heart beats calmly and normally" or "I have a calm and steady pulse"

(Pause - a moment of silence is allowed)

Repeat once to yourself: "I am completely calm and relaxed"

(Pause - a moment of silence is allowed)

Maintain awareness of your calm, nasal, and silent breathing.

→ The goal is not to modify the heart rate but rather to be aware of it.

Breathing exercise (breathing control is given)

Repeat 5 times to yourself: "My breathing is calm and balanced"

(Pause - a moment of silence is allowed)

Repeat once to yourself: "I am completely calm and relaxed"

(Pause - a moment of silence is allowed)

Maintain awareness of your calm, nasal, and silent breathing.

→ The aim is to achieve a free and harmonious breathing pattern.

Exercise of the solar plexus (belly button area)

Repeat 5 times inwardly to yourself: "My belly is warm and radiates heat"

(Pause - allow a moment of silence)

Repeat 1 time inwardly to yourself: "I am completely calm and relaxed"

(Pause - allow a moment of silence)

Keep your awareness on calm, nasal, and silent breathing.

Exercise of the forehead (head)

Repeat 5 times inwardly to yourself: "My forehead is pleasantly cool"

(Pause - allow a moment of silence)

Repeat 1 time inwardly to yourself: "I am completely calm and relaxed"

(Pause - allow a moment of silence)

Keep your awareness on calm, nasal, and silent breathing.

**3rd Stage - Guided Imagery for Self-Promotion of Health and Personal Power**

After the person is in a deeper state of relaxation, we will "plant the seed" of self-promotion of health and personal power.

"Now that you feel completely calm, relaxed, and safe, as you inhale, imagine the air entering your system. By bringing more oxygen, energy, and vitality to each of your cells, organs, and systems, you also bring love, peace, well-being, joy, harmony, strength, health, and healing, awakening your personal power, your inner wisdom. Imagine your body being nourished with a golden light that emanates energy, healing, and love. As you exhale, imagine that you are expelling everything that imprisons, tires and wears you out, what you no longer want to keep in your life. Feel more loose, more free, happier. (Repeat this instruction 3 times)

Say to yourself: "I am calm and relaxed, I love myself and I know who I am, I have value, I am unique and incomparable. I am everything I want to be. I am, I am, I am. I accept myself as I am, with qualities and flaws, good experiences and not so good experiences, I like myself as I am and every day I work towards a better version of myself and allow myself to grow as a person". Enjoy a little more of this state of harmony that you have created within yourself. Stay with yourself, gather yourself and let yourself stay, trust. Allow yourself to travel where your soul wants to take you. Accept everything you can experience, with tenderness, without questioning. (After this instruction, allow a moment of silence). Now, to remember the journey you were able to lead within yourself, to record that it really happened, take a picture of the most special moment you lived and keep that picture as a memory of that journey, in a secret place within yourself, that only you know where it is".

**Finalization - Exiting the relaxation state...**

"It is time to return, bringing with you all the good experiences you had. Thank yourself for allowing yourself to relax and travel within yourself. Bring with you the treasure you discovered inside yourself, knowing that you can use it whenever you want, in the place that suits you best. Slowly prepare to return to the here and now, to the space you are in. The journey back will be guided by the five senses.

Begin by awakening your hearing, paying more attention to my voice and the sounds around you. Awaken your taste by swallowing saliva and moistening your lips. Release your sense of smell by feeling the scents around you. Awaken your sense of touch by feeling more and more of your body, the contact with the chair, feeling your feet on the floor, legs, hips and buttocks, feeling your arms and hands opening and closing, moving your legs, massaging your legs and knees, stretching, thus preparing to open the "windows" to the outside again. Slowly open your eyes when you are ready. Embrace yourself and thank yourself for the journey you allowed yourself to take."

**Figure S1**

*CONSORT Flow Chart.*

Analyzed (n= 44)

Excluded (n= 0)

Analyzed (n= 34)

Excluded (n= 0)

Analyzed (n= 28)

Excluded (n= 0)

Lost to follow-up (n= 0)

Lost to follow-up (n= 0)

Discontinued intervention (n= 0)

Lost to follow-up (n= 0)

Discontinued intervention (n= 0)

Allocated to passive control group (n= 44)

Allocated to active control group (n= 44)

♦ Received allocated intervention (n= 34)

♦ Did not receive allocated intervention (n= 10)

Allocated to intervention (n= 45)

♦ Received allocated intervention (n= 28)

♦ Did not receive allocated intervention (n= 17)

Excluded (n= 0)

Randomized (n= 133)

Assessed for eligibility (n= 133)

**Figure S2**

*Estimated marginal means for Depression, Anxiety, and Stress estimated under time*condition model.*


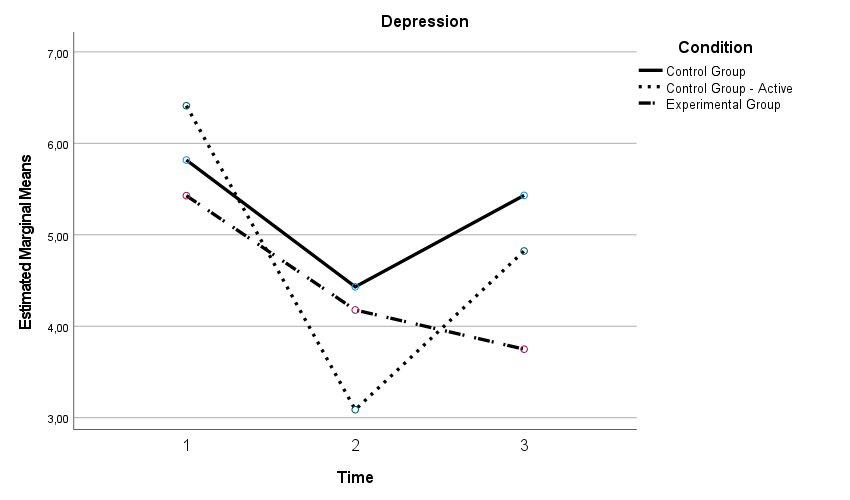


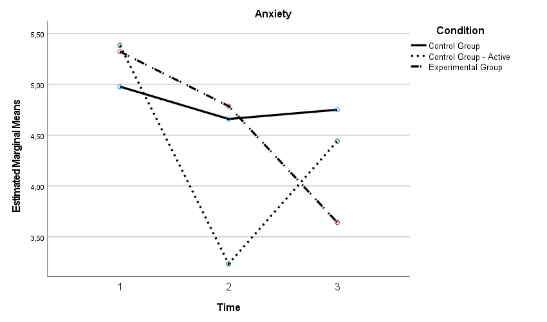


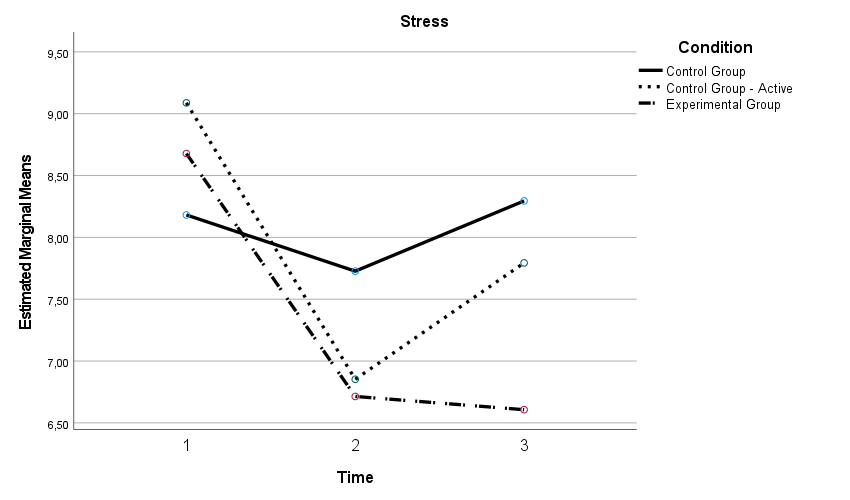


**Figure S3**

*Estimated marginal means for Self-compassion, Self-concept, and Emotion Regulation estimated under time*condition model, respectively.*


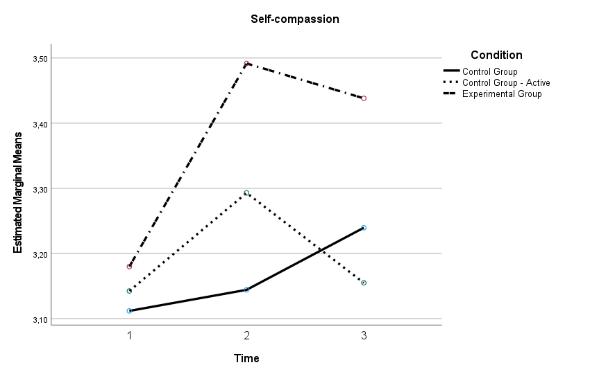


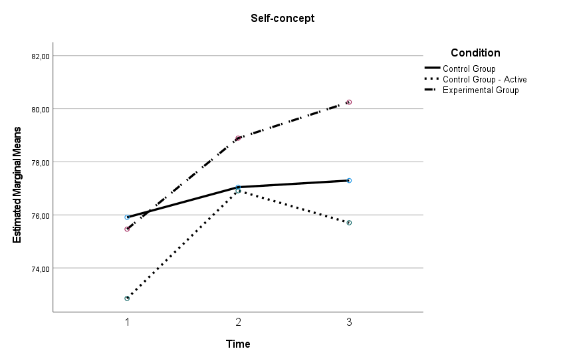


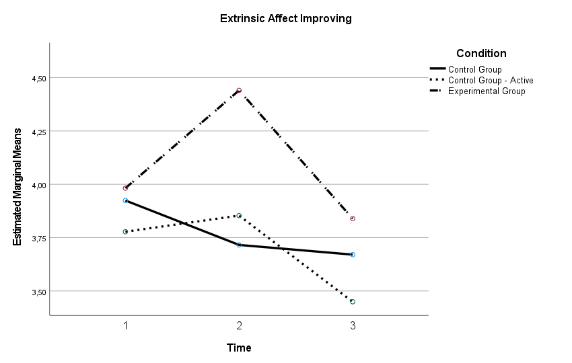


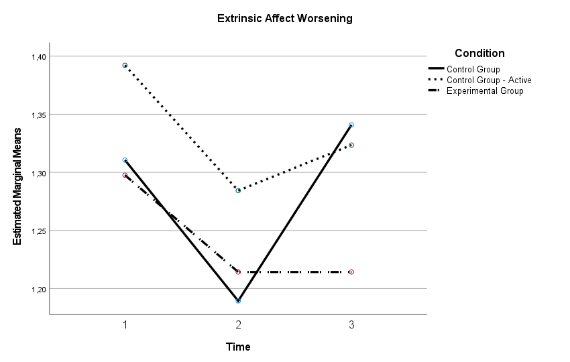


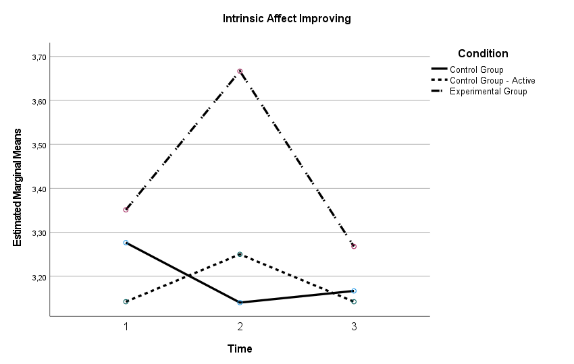


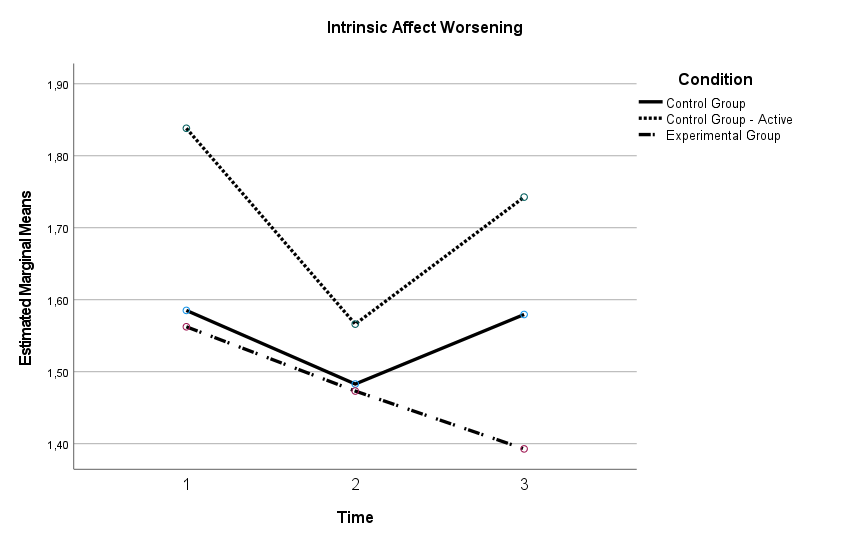


**Figure S4**

*Estimated marginal means for Wellbeing Dimensions and Subjective Happiness estimated under time*condition model.*


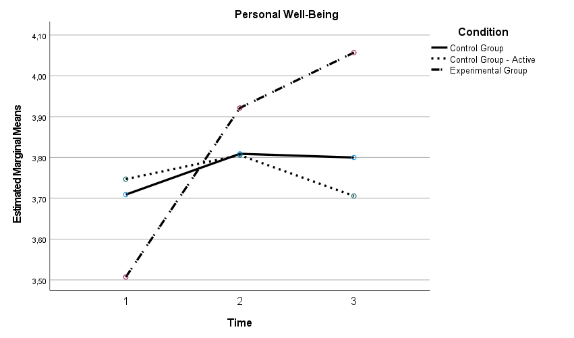


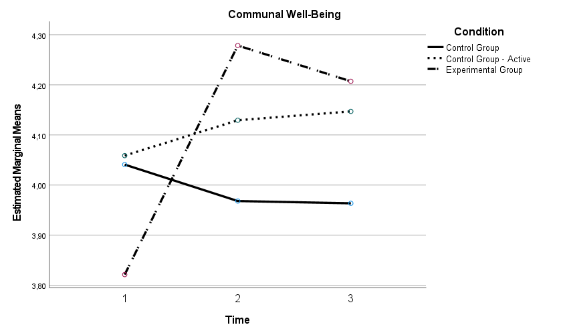


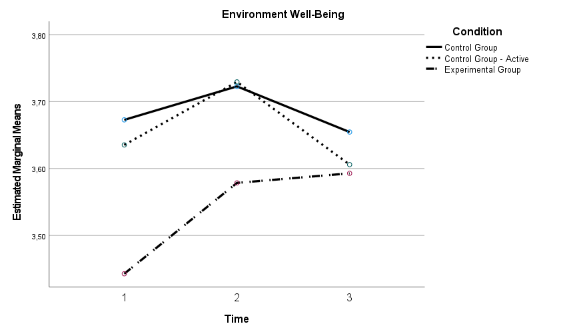


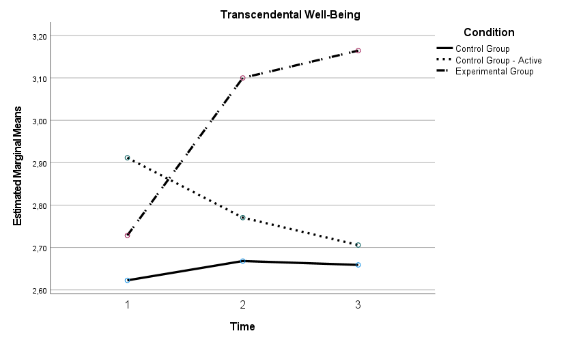


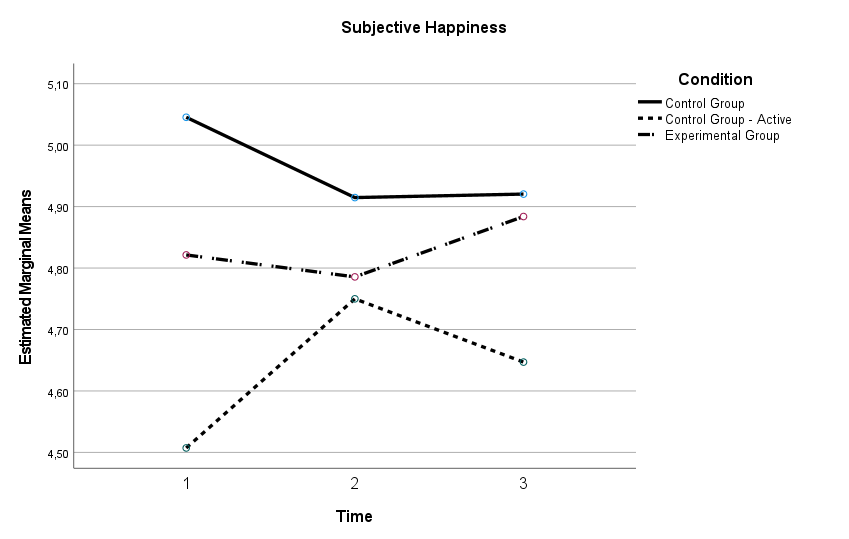

Supplement: sj-docx-1-hpq-10.1177_13591053231220710 – Supplemental material for Effect of online Kundalini Yoga mental health of university students during Covid-19 pandemic: A randomized controlled trial [file sj-docx-1-hpq-10.1177_13591053231220710.docx]
